# Complete Genome Sequences of Three *Bacillus anthracis* Bacteriophages

**DOI:** 10.1128/genomeA.01164-17

**Published:** 2018-01-04

**Authors:** Sivan Alkalay, Sarit Sternberg, Shunit Coppenhagen-Glazer, Ronen Hazan

**Affiliations:** a Faculty of Dental Sciences, Hebrew University, Hadassah School of Dental Medicine, Jerusalem, Israel

## Abstract

The new highly effective *Bacillus anthracis* phages Negev_SA, Carmel_SA, and Tavor_SA were isolated from soil samples, and their complete genomes were sequenced and analyzed. The isolated phages have potential use in future phage therapy treatment against anthrax.

## GENOME ANNOUNCEMENT

*Bacillus anthracis* is a Gram-positive spore-forming bacterium that causes the anthrax disease (1, 2). *B. anthracis* is considered one of the most dangerous bioterrorism agents due to its remarkable survival abilities and its high lethality (3). Although antibiotics are usually effective against *B. anthracis* infections (4), antibiotic resistance is regarded as a threat (5). Moreover, it is expected that in the event of a bioterrorism attack, antibiotic-resistant strains will be deployed. A possible solution to this threat is to have an arsenal of lytic bacteriophages that are effective against *B. anthracis*. Yet, so far, only 12 of them specifically attack *B. anthracis* strains (6).

Here, we report the isolation of three new *B. anthracis* phages, Negev_SA, Carmel_SA, and Tavor_SA, which could be developed to treat anthrax infections.

The phages were isolated from soil samples from various places in Israel, mainly the Golan Heights region, where several outbreaks of anthrax disease were reported. Purification was conducted using the phage titration method, as previously described (7), with a few modifications. Briefly, samples were incubated in LB broth for a few days, followed by centrifugation at 2,650 × *g* for 10 min and filtration through filters with 0.22-μm pores. Two hundred microliters of exponentially grown bacterial cultures (10^8^ CFU/ml) was added to 3.5 ml of 0.5% agarose, which was poured onto an LB plate. Three microliters of each sample was spotted on the bacterial lawn, and the plates were incubated overnight at 37°C.

The phages’ DNA was purified using a phage DNA isolation kit (Norgen Biotek), libraries were prepared with an Illumina Nextera XT DNA kit (San Diego, CA), and sequencing was performed using the Illumina MiSeq platform. The quality of the 150-bp paired-end reads was assessed with FastQC (https://www.bioinformatics.babraham.ac.uk/projects/fastqc). The *de novo* assembly with the trimmed paired-end reads was performed using Geneious version 10 (Biomatters). The mean coverages are 30.7× (±10.4) for Negev_SA, 35.2× (±11.4) for Carmel_SA, and 31.3× (±11.6) for Tavor_SA. Annotation was performed with PHAST (PHAge Search Tool). Analysis of the open reading frames and phylogenetic tree generation were performed with Geneious version 10 and its plugins.

The genomes of Negev_SA, Carmel_SA, and Tavor_SA are linear and contain 40,375 bp, 40,165 bp, and 40,397 bp, respectively. The G+C contents of Carmel_SA and Tavor_SA (both 35.2%) and Negev_SA (34.9%) are similar to that of *B. anthracis* (35.1%).

Carmel_SA, Tavor_SA, and Negev_SA are similar to *Bacillus* phages Gamma (GenBank accession number NC_007458) and Fah (DQ222851) belonging to the *Siphoviridae* family of the order *Caudovirales*. There are 57 coding sequences in Negev_SA, 56 in Carmel_SA, and 61 in Tavor_SA. The genes of the three phages are similar, except for a few exceptions; Carmel_SA is the only genome which contains beta-galactosidase and does not code for a phage terminase small subunit. Only Negev_SA has a flagellar hook-length control protein (FliK) and a phage antirepressor.

The phages contain repressor proteins, site-specific recombinases, and antirepressor proteins, which indicates that these phages have lysogenic capabilities that might impair the use of these phages for therapy. However, the fact that they are genetically related to phage Gamma, which is lytic (8), and that in our experiment the phages exert clear plaques and dramatic killing in liquid, perhaps indicate that they might still be candidates for therapy.

### Accession number(s).

The complete genome sequences of *B. anthracis* phages Negev_SA, Carmel_SA, and Tavor_SA are available in GenBank under the accession numbers KY963370, KY963371, and KY963369, respectively.

## References

[1] Sweeney DA, Hicks CW, Cui X, Li Y, Eichacker PQ 2011 Anthrax infection. Am J Respir Crit Care Med 184:1333–1341. doi:10.1164/rccm.201102-0209CI. 21852539 PMC3361358

[2] Spencer RC 2003 Bacillus anthracis. J Clin Pathol 56:182–187. doi:10.1136/jcp.56.3.182. 12610093 PMC1769905

[3] Klietmann WF, Ruoff KL 2001 Bioterrorism: implications for the clinical microbiologist. Clin Microbiol Rev 14:364–381. doi:10.1128/CMR.14.2.364-381.2001. 11292643 PMC88979

[4] Jamie WE 2002 Anthrax: diagnosis, treatment, prevention. Prim Care Update OB/GYNS 9:117–121. doi:10.1016/S1068-607X(02)00100-2.

[5] Athamna A, Athamna M, Abu-Rashed N, Medlej B, Bast DJ, Rubinstein E 2004 Selection of *Bacillus anthracis* isolates resistant to antibiotics. J Antimicrob Chemother 54:424–428. doi:10.1093/jac/dkh258. 15205405

[6] Gillis A, Mahillon J 2014 Phages preying on *Bacillus anthracis*, *Bacillus cereus*, and *Bacillus thuringiensis*: past, present and future. Viruses 6:2623–2672. doi:10.3390/v6072623. 25010767 PMC4113786

[7] Khalifa L, Brosh Y, Gelman D, Coppenhagen-Glazer S, Beyth S, Poradosu-Cohen R, Que YA, Beyth N, Hazan R 2015 Targeting *Enterococcus faecalis* biofilms with phage therapy. Appl Environ Microbiol 81:2696–2705. doi:10.1128/AEM.00096-15. 25662974 PMC4375334

[8] Davison S, Couture-Tosi E, Candela T, Mock M, Fouet A 2005 Identification of the *Bacillus anthracis* γ phage receptor. J Bacteriol 187:6742–6749. doi:10.1128/JB.187.19.6742-6749.2005. 16166537 PMC1251577

